# Peat Pellets as Enrichment Material for Weaned Piglets

**DOI:** 10.3390/ani16203214

**Published:** 2026-10-13

**Authors:** Anouschka Middelkoop, Hannele Kettunen, Anne Maria Stevina Huting, Juhani Vuorenmaa, Francesc Molist

**Affiliations:** 1 Schothorst Feed Research B.V., 8218 NA Lelystad, The Netherlandsfmolist@schothorst.nl (F.M.); 2 Hankkija Oy, FI-05880 Hyvinkää, Finland; hannele.kettunen@hankkija.fi (H.K.);

**Keywords:** behaviour, biting, edible, enrichment, faecal consistency, post-weaning, substrate, tail

## Abstract

We aimed to explore the effect of 8 mm peat pellets as enrichment material for weaned piglets. A total of 288 piglets were allocated to one of two treatments: peat pellets (PPs) or chopped straw (CS) as edible enrichment. Twelve pigs were housed per pen, with 12 weaner pens per treatment. Pigs were weighed at day 0, 9, 14, and 28 post-weaning. Faecal consistency was recorded 3 times a week. Piglet behaviour was observed at days 8 and 18, and damage on ears and tail were scored weekly. More cups of edible enrichment were provided to PP pens than to CS pens between day 0–14 to ensure ad libitum availability (*p* = 0.04). On day 18, PP piglets showed greater exploratory interaction with the edible enrichment than CS piglets (*p* = 0.045). PPs improved performance in the early post-weaning period, resulting in a higher initial feed intake (*p* = 0.0498) and growth (*p* = 0.007) of newly weaned piglets between days 0 and 9, and improved faecal scores between days 14 and 28 (*p* = 0.007). At the end of the study, piglets had improved body weight uniformity (*p* = 0.04) and tended to have a lower prevalence of tail damage (*p* = 0.07) when provided with PPs versus CS. In addition, PP piglets tended to spend less time on ear biting (*p* = 0.06) on day 8 than CS piglets. PPs are proposed as suitable enrichment material and were associated with improved zootechnical performance and faecal consistency in weaned piglets.

## Simple Summary

Tail damage and intestinal dysbiosis are significant welfare concerns for weaned piglets. Gut health-promoting feed materials may serve a dual purpose by improving zootechnical effectiveness and, when suitably delivered, stimulating exploratory behaviour in piglets to enhance welfare. The post-weaning provision of peat as 8 mm pellets allowed for exploration and play towards the pellets. Numerical reductions in ear-biting behaviour and tail damage were observed compared with chopped straw, although these findings were exploratory, and the overall incidence of biting-related outcomes was low in both treatment groups. The presence of peat pellets instead of chopped straw stimulated initial feed intake and growth. Towards the end of the weaner phase, it improved body weight uniformity and faecal consistency. This study showed that peat pellets can be considered suitable enrichment material for weaned piglets and can exert beneficial effects on faecal consistency and zootechnical performance.

## 1. Introduction

Weaning of piglets is a stressful event with many changes, including social, environmental, and dietary stressors. In general, these stressors result in a decreased feed intake during the first days post-weaning [1] and in an increased level of manipulative behaviour towards the abdomen, ears, and tails of pen mates [2]. The low feed intake and stress damages the intestinal wall and compromises piglet immune function, resulting in gastrointestinal inflammation, gut microbiota dysbiosis, and reduced growth [3,4,5]. To have a positive impact on sustainability of pig production, it is important to support piglet resilience around weaning towards the variety of microbial, physical, and physiological stress factors by nutritional as well as management strategies.

Pigs spend a significant amount of their time on oral exploration of their environment by nosing, rooting, and chewing. This behaviour can be re-directed to pen mates, especially in barren versus enriched housing systems (reviewed by [6]) and on farms rearing pigs with intact or long tails versus shortly docked tails [7,8]. Moreover, tail biting has been linked to poor health, in which health challenges could be either the result or cause of tail biting [9]. The minimum standards for rearing pigs are laid down in regulations to protect pig welfare. In European Council Directive 2008/120/EC, the EU Directive on the protection of pigs [10], it is stated that pigs must have permanent access to a sufficient quantity of material to enable proper investigation and manipulation activities, such as straw, hay, wood, sawdust, mushroom compost, peat, or a mixture of such, which does not compromise the health of the animals. Additionally, the Directive states that tail-docking is only allowed with approval of the veterinarian and if there is evidence that injuries to other pigs’ tails have occurred on the farm. In 2016, the European Commission issued a recommendation regarding management of tail biting in pigs, clarifying that enrichment materials should be edible, chewable, investigable, and manipulable and should sustain the interest of the pigs [11]. Their working document on best practices with a view to the prevention of routine tail-docking in pigs also suggested that treated peat can be seen as an essential component of pig enrichment when it is used in combination with other enrichment materials [12].

Peat is a natural material and a source of bioactive compounds [13]. Peat has been considered as a functional fibre source in the diet with promising effects for the gut health and performance of animals, with observed effects on mycotoxin binding, modulation of microbial population and fermentation (advantages over lignocellulose), diarrhoea, growth, and feed conversion [14,15,16,17]. When provided to pigs in the application form of large peat pellets, practical experience not only suggested good palatability and a lower diarrhoea frequency but also reported a lower tail biting prevalence in piglets with access to peat pellets [18]. While effects on pig behaviour have been reported for loose, non-pelletized peat, corresponding scientific data for peat pellets are not available. The provision of loose peat in a tray versus no enrichment significantly increased the time piglets’ spent on foraging and play behaviour while reducing manipulation and fighting within the weaner pen. When peat was provided from the pre-weaning phase onwards, it was also observed to improve post-weaning growth [19]. A similar effect was observed when enrichment substrate was provided on the floor [20]. Loose peat and the combination of the four rooting materials had the largest effect on inducing positive behaviours (i.e., exploration and play) and reducing negative social behaviours (i.e., aggression and manipulation) versus silage or straw [20]. The way gut health-promoting feed materials are provided can therefore be adapted to stimulate exploratory behaviour in piglets and to function as enrichment material. The current study aims to explore the effect of peat pellets on the behaviour and performance of weaned piglets. We hypothesised that peat pellets would increase exploratory and play behaviour, reduce manipulative behaviours directed towards pen mates and associated body damage, and enhance zootechnical performance and faecal consistency in weaned piglets.

## 2. Materials and Methods

The experiment was conducted at the Schothorst Feed Research (SFR, Lelystad, The Netherlands) weaner pig facility in the period from end of April 2025 until the beginning of July 2025. The experiment was designed and conducted to cause minimum suffering or distress to the animals consistent with the scientific objectives and in accordance with the restrictions provided under Animal and Human Welfare Codes/Laboratory practice codes in The Netherlands. During the study, appropriate animal health and welfare inspections were carried out. The use of the test product was accepted (certificate number V251035) by IKB Varken of Kiwa VERIN B.V (Nieuwegein, The Netherlands).

### 2.1. Experimental Design

The experiment was performed during the first 28 days post-weaning, and set-up as a complete randomised block design with two treatment groups and 12 pens per treatment. The experimental treatments were: (1) Peat pellets (PP): piglets provided with ProHumi^®^ Farm in pellet form as an edible enrichment material; and (2) Chopped straw (CS): piglets provided with chopped straw as edible enrichment material. An overview of the treatments is presented in Table 1.

The peat pellets were 8 mm in diameter and 10–20 mm in length, and they had a volume weight of 640 g/L. ProHumi^®^ Farm peat pellets (Hankkija Oy, Hyvinkää, Finland) contained approximately 19% crude fibre, 70% total dietary fibre, and around 30% humic acids. The composition and chemical properties of the peat pellets can be found in Supplementary Table S1. Microbiological safety was ensured during the double heat-treatment of the production process by reaching temperatures > 81 °C. The peat was derived from Finnish peatlands. Chopped straw, chopped to a length of about 2–2.5 cm, was purchased from ABZ Diervoeding (Nijkerk, The Netherlands), and had a volume weight of 80 g/L.

**Figure 1:**
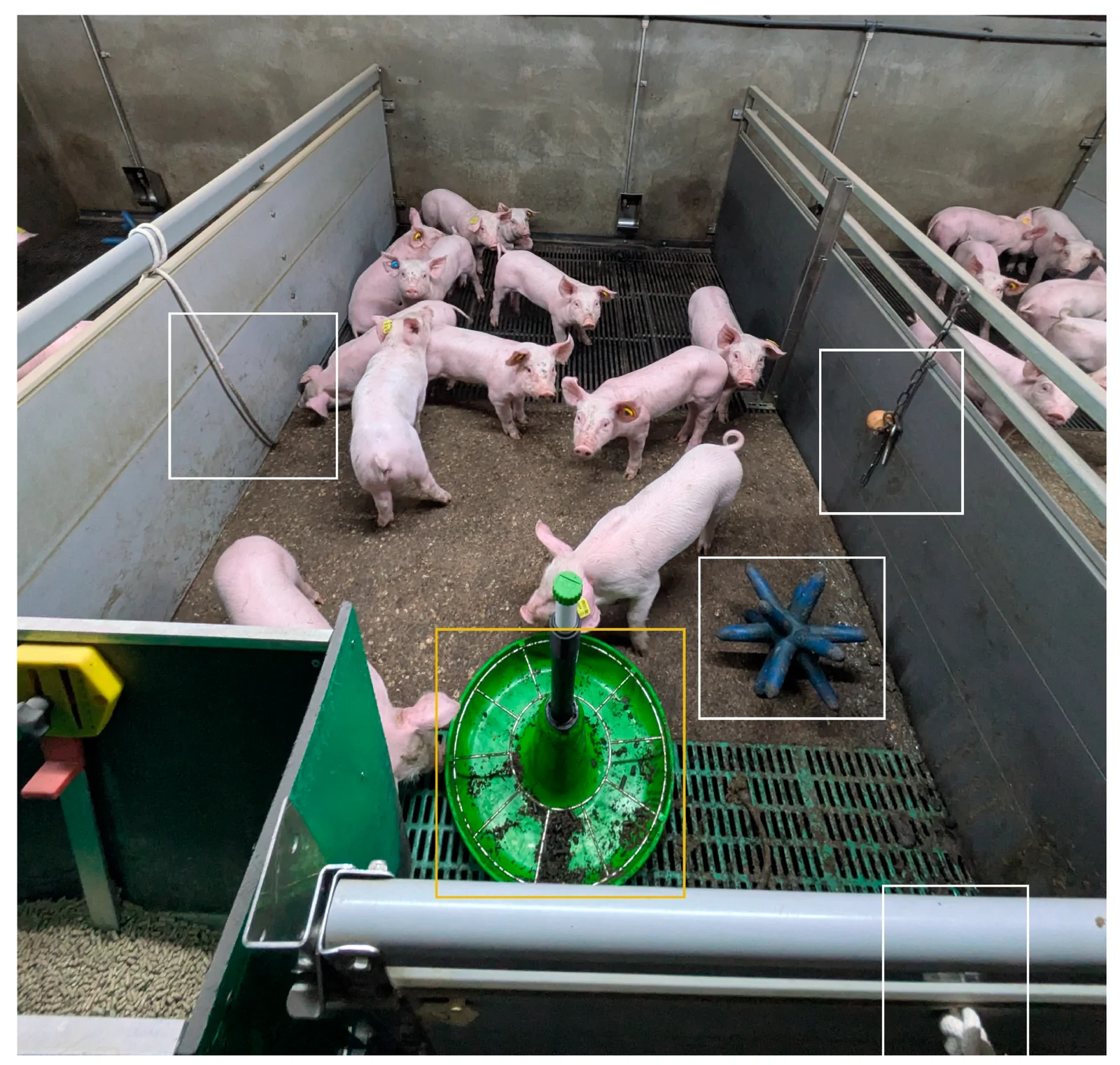
The experimental housing conditions and location of the edible enrichment (brown box) and standard enrichment (white boxes) within the pen.

Per pen, piglets had access to two cotton ropes (MS Schippers, Hapert, The Netherlands; one attached in the front and one halfway), a floor-based rubber toy (Easyfix Luna, MS Schippers, Hapert, The Netherlands), and a metal chain with a solid ball and HDPE plastic rod (or solely its remaining metal pin, standardised per room; MS Play Material, MS Schippers, Hapert, The Netherlands). These enrichment materials are collectively referred to as standard enrichment material (Table 1). In addition to the standard enrichment materials, a plastic feeding bowl (MS Clickfeeder Maxi 9L; MS Schippers, Hapert, The Netherlands) with 10 feeding places was placed in the pen, attached to the slats at the transition from slats to solid floor (Figure 1). In the plastic feeding bowl, either PPs or CS were provided, dependent on the treatment group. These enrichment materials are collectively referred to as edible enrichment material. Fresh amounts of edible enrichment material were provided daily to each pen in early morning and mid-afternoon to provide the material ad libitum. The provisioning dosage was noted as the number of cups per pen provided and increased if only a limited amount of material was left.

### 2.2. Animals and Housing

The study included 67 TN70 sows (mean parity 4.3 ± 0.25; range 1–8) inseminated with semen from 14 different TN Tempo boars. Piglets (TN Tempo × TN70) originating from these litters were followed throughout lactation, and subsequently a subset of 288 piglets, were selected for the study at weaning. Sows and piglets were housed, fed, and managed according to standard EU (EU Directive 98/58/EC [21] and 2008/120/EC [10]) and Dutch farming practices. At birth, piglets were ear tagged and weighed, and their sex was determined and registered. No teeth clipping, tail docking, or castration were performed. Within 2 days after birth, litter size was standardized to 14–15 piglets per litter through cross-fostering. Piglets received an injectable iron supplement (1 mL, Iron-ject^®^; Dopharma, Raamsdonksveer, The Netherlands) at 3–4 days of age. Piglets were not exposed to vaccinations, except that their sows were vaccinated according to the manufacturer’s vaccination scheme with an inactivated vaccine against neonatal colibacillosis and *Clostridium* infections (SUISENG^®^, HIPRA, Amer, Girona, Spain). Farrowing pens measured 2.50 × 2.66 m, providing approximately 5 m^2^ for loose sow housing. To reduce the risk of piglet crushing, sows were temporarily confined in a farrowing crate (2.24 × 0.65 m, including balance floor) during the first 5 days postpartum. The pen flooring consisted of plastic slats and included a heated solid-floor creep area for piglets. Each pen was equipped with a drinking nipple and feed trough for the sow, and a shielded creep area, a drinking nipple, and creep feeder for the piglets. Piglets received a commercial solid diet (Speenkorrel; ABZ Diervoeding, Leusden, The Netherlands) from 1 week of age onwards. Each farrowing pen housed a sow, and her litter and was provided with two enrichment materials: a cotton rope (MS Schippers, Hapert, The Netherlands) and a floor-based rubber toy (Easyfix Luna; MS Schippers, Hapert, The Netherlands).

At weaning (day 30.1 ± 0.08 of age; range 28–34 days of age), piglets were individually weighed and moved from the farrowing to weaner rooms. Piglets showing signs of ear or tail damage, injury or illness at the time of weaning were excluded from selection. After being included in the study, piglets were excluded if a piglet needed to be moved to a hospital pen for proper recovery, found dead, or reached a humane end point. Humane endpoints were defined as clinical illness without a reasonable prospect of recovery, in which case the animals were humanely euthanized via intracardiac injection with T61^®^ (REG NL 5356; Intervet Nederland B.V., Boxmeer, The Netherlands) after sedation with Zoletil^®^ (REG NL 115916; Virbac Laboratories, Carros, France).

The experiment was conducted in two weaning batches with 3 weeks in between. Four rooms were used for the experiment, with two rooms per batch and three replicates per room. A replicate consisted of a pair of pens (one PP pen and one CS pen) located within the same room. Consequently, each batch comprised six replicates and the experiment comprised 12 replicates in total, providing 12 pens per treatment. The same selection procedures and housing conditions were applied in both batches of the experiment. A computer system randomly allocated the pens to treatments, and the exact layout of the treatments in each weaner room is included in Supplementary Figure S1. As a method of blinding, numerical coding was used and each pen was identified with a treatment code. However, since the differences between treatments were visible, complete blinding could not be achieved. A randomization process was performed to allocate a total of 288 piglets to replicates while balancing gender (both genders) and weaning weight (8.27 ± 0.032 kg; range 7.00–9.15 kg). In addition, piglets from the same sow were distributed across treatments and replicates as evenly as possible. Among the sows contributing more than one piglet to the study (i.e., 62 out of 67 sows), piglets from 47 sows were represented in both treatments, while the PP and CS treatments included piglets originating from 56 (parity 4.2 ± 0.28) and 58 sows (parity 4.2 ± 0.27), respectively.

Piglets were housed in groups of 12. Each pen contained six male and six female piglets originating from 8 to 11 different litters. Pens measured 1.60 × 2.59 m, providing 0.34 m^2^ per piglet, and had partially slatted floors and solid plastic pen partitions (Figure 1). The pens were equipped with a feeder with three feeding places and a drinking nipple. All rooms were windowless and climate controlled. Room temperature was automatically regulated by a climate computer following a temperature curve targeted at 29 °C at the day of weaning to 23.4 °C at 28 days post-weaning. The rooms were ventilated with outdoor air. Humidity in the rooms was dependent of outdoor humidity and ventilation rate. Artificial lights (LED) were provided from 5:00 till 21:00. The transition from lights off to on and vice versa was through a twilight period. Piglets had ad libitum access to feed and water.

Commercial weaner I (“Speenkorrel”, ABZ Diervoeding, Leusden, The Netherlands) and weaner II diets (“Biggenkorrel”, ABZ Diervoeding, Eindhoven, The Netherlands) were fed in the trial. The commercial diets were pelleted at 3 mm diameter. Weaner I was fed from days 0–14 post-weaning and weaner II was fed from days 14–28 post-weaning. The dietary change at day 14 was abrupt. The weaner I diet contained approximately 15.8% crude protein, 4.7% crude fibre, 8.2 MJ net energy, and 11.2 g standardised ileal digestible (SID) lysine/kg. Piglets received this diet also in the pre-weaning phase, from about 5–7 days of age. The weaner II diet contained approximately 17.7% crude protein, 3.7% crude fibre, 9.7 MJ net energy, and 12.3 g SID lysine/kg. Zootechnical additives added to the diets included phytase, beta-glucanase, and α-xylanase, and for the weaner I diet, benzoic acid was added. The diets were based on wheat, maize, soybean meal, and barley.

### 2.3. Measurements

The selection process and trial measurements were performed by different personnel, respectively. Piglets were inspected daily by trained animal care staff as part of routine welfare monitoring to identify piglets requiring medical treatment or biting interventions. Health assessments included evaluation of general appearance, body condition, locomotion, and the presence of visible abnormalities such as injuries, watery diarrhoea, or signs of disease. Deviations from the normal health status were recorded. In case (antibiotic) treatment was necessary, the individual piglet number, the type of treatment, and treatment duration were recorded. Injectable antibiotics were applied as soon as clinical signs were detected.

Individual piglet body weight was recorded at weaning (day 0), and on days 9, 14, and 28 post-weaning. Body weight uniformity was expressed as the coefficient of variation (CV). Average daily gain (ADG, g/piglet/d) was calculated on pen basis between days 0–14, days 14–28, and days 0–28 post-weaning. This was done using body weight gain divided by the number of piglet days. Feed intake (i.e., allowance and refusal) per pen was recorded between days 0–14 and 14–28 post-weaning, and average daily feed intake (ADFI, g/piglet/d) was calculated for each phase, as well as the total experimental period considering the number of piglet days. The feed conversion ratio (FCR) was calculated as ADFI divided by ADG on pen basis between days 0–14, 14–28, and 0–28.

The amount of PP and CS provided was recorded on a daily basis. As material lost through the slatted floor could not be quantified, actual intake could not be determined. In the early morning and mid-afternoon, fresh amounts of PP and CS were provided in each pen. A fixed minimum amount of one cup in the morning and one cup in the afternoon was provided in each pen. One cup represented on average 86.6 g of PP and 10.5 g of CS. If, during the next provisioning moment, only a limited amount of material was left (determined on pen basis), the provisioning dosage was increased for that pen to provide the material ad libitum. The number of cups provided was recorded on pen level on a daily basis. The amount of material provided (g) was calculated per pen based on the number of cups and the average cup weight, and average daily substrate provision (ADSP) was expressed as g/piglet/d based on the number of piglet days.

Faecal consistency was measured per pen three times per week between days 0–28 post-weaning at an 8-point scale (ranging from score 2 to 9) from severe water thin diarrhoea to hard, dry, and lumpy faeces, with a score of 6 indicating normal faecal consistency, as described by Guan et al. [22] and averaged weekly as well as for days 0–14, 14–28, and 0–28. The faecal consistency protocol can be found in Supplementary Table S2.

The prevalence of ear and tail damage was scored once a week at days 7, 14, 21, and 28 using the following three-point scale according to Huting et al. [23]; score (0) no damage, score (1) bite marks, superficial scratches, injuries with fresh or dried blood, or redness/swelling, and score (2) incomplete, with a part of the ear or tail missing. Ears and tails were scored separately. The number of piglets belonging to each of the damage scores was recorded by entering the pen and observing the ears and tail of each pig according to the scoring system.

In case of ear or tail damage, climate, feed and water systems were inspected for potential irregularities. Biting interventions, i.e., additional enrichment and removal of biter or victim, were recorded on pen level. In case of the presence of fresh blood on ears or tail(s) of one or more piglets within the pen, a jute bag was added to the pen. This scenario was defined as manipulative biting within the pen. In case a piglet was observed with ear or tail score 2, it was considered a victim and was moved to a hospital pen for recovery. When more than one piglet was observed with ear or tail score 2, there was considered to be a persistent biter within the pen, and the biter was removed from the pen. In both victim or biter scenarios, a jute bag was also added to the pen.

Behavioural observations were done using 1.5 min. instantaneous scan sampling for four sessions of 1 h/d, i.e., 160 scans/piglet/day, to observe play, explorative, and damaging behaviours of weaned piglets. Behavioural observations were performed at day 8 and day 18 post-weaning on trial days without any other measurements. Observation sessions started at 8:15 h, 9:30 h, 14:15 h, and 15:30 h. Piglets were habituated to the observers walking by the pens before the first observation day to prevent piglets being startled during the observations. The behaviours were scored by three well-trained observers, which scored the behaviours from the corridor adjacent to the pens. Observers were balanced across treatments, observation times, and changed rooms every hour. The number of piglets in the pen involved in the behaviours in the ethogram were recorded per scan, as well as the total number of piglets present in the pen. The ethogram was based on Middelkoop et al. [24,25] and is given in Table 2. The ethogram focused on pre-defined behaviours of interest rather than a complete time budget of all behaviours performed by the piglets. Behavioural categories were scored as mutually exclusive. Behavioural states in the weaner pen were expressed as proportions of time spent on that behaviour by piglets in the pen.

### 2.4. Statistical Analysis

Pen data were corrected for any piglet removed from the study based on the number of animal days (number of animals x number of days). The number of piglets performing a specific behaviour was divided by the total number of piglets in that pen to calculate the percentage of piglets performing that behaviour for each observation. Subsequently, the percentage of observations that a certain behaviour was recorded was calculated per pen per observation day. Before analyses of the effect of edible enrichment type on the number of biting interventions, the interventions that concerned removal of a victim piglet with meningitis in the pen were excluded.

The experimental data were analysed using GenStat^®^ Version 24 for Windows™ (VSN International Ltd., Hemel Hempstead, UK). Observations were marked as outlier if the absolute residual (fitted–observed value) exceeded 2.5 × standard error of the residuals of the dataset. The number of observations identified as outliers ranged from 0 to 5.6% depending on the variable. All observations identified as outliers are highlighted in the Supplementary Dataset. Missing values were estimated using least square estimates. If at least one of the performance response parameters, i.e., feed intake, body weight gain, or feed conversion ratio, was identified as outlier, all three parameters of that experimental unit in that measurement period were designated as missing value. Outlier screening was restricted to continuous variables. Baseline variables (BW and CV in BW at day 0 post-weaning) were not screened because they represented pre-treatment characteristics used for the allocation of piglets to experimental units. Analyses on the complete dataset, including outliers, were performed as a sensitivity analysis. Where results differed between the complete-data and outlier-excluded analyses, these differences are reported in the Section 3.

The study is a randomised complete block design, with pen as the experimental unit for statistical purposes to determine efficacy on zootechnical performance (BW, CV in BW, ADG, ADFI, FCR, faecal consistency) and the effect on piglet behaviour. Data were analysed using one-way analysis of variance (ANOVA) via the General Linear Model function. The models included treatment as fixed effect and batch, room nested within batch, and replicate nested within room within batch as random effect. The normality of the model residuals was assessed using the Shapiro–Wilk test and visual inspection of residual plots. Homogeneity of variances was assessed using Bartlett’s test. Behavioural data were transformed using square root prior to statistical analyses to meet the assumption of normally distributed residuals. Feed-related fighting did not achieve normality of residuals after transformation and was therefore excluded from further analysis. Significant differences are declared at *p* ≤ 0.05, with near significant trends as 0.05 < *p* ≤ 0.10. Data are presented as absolute means and standard error of the mean (SEM), except for damage score data, which are presented as model-predicted probabilities (%) for each damage score category within a treatment, together with their associated 95% confidence intervals (CI). Damage scores consisted of category counts per pen and were analysed separately for each experimental day using ordinal regression, with pen as the experimental unit. Damage score data were analysed using a generalised linear model (GLM) with a multinomial distribution and a cumulative logit link function. The model included treatment as a fixed effect and replicate as a blocking factor. The significance of treatment effects was assessed after accounting for replicate effects in the model. Reported *p*-values are based on the GLM analysis.

## 3. Results

### 3.1. Medical Treatments, Mortality, and Removals

Morbidity (2.1%), mortality (0.7%), and removals (1.0%) were not analysed across treatments. A total of 288 piglets entered the experiment at weaning. Of these 288, six piglets (2.1%) were medically treated. Medical treatments consisted of antibiotics (i.e., Depocilin REG NL 4259/Dexa-ject^®^ 2 REG NL 110424/Dofatrim REG NL 8762), given in combination with a painkiller (Melovem^®^ 5 REG NL 103350). Medical treatments were related to arthritis (two PP and one CS piglet), meningitis (one PP and one CS piglet), and diarrhoea (one PP piglet). Three piglets, including two previously treated piglets, were removed from the trial (one PP piglet at day 11 post-weaning and two CS piglets at day 25 post-weaning) and moved to a hospital pen for further treatment due to clinical signs of meningitis, increasing their risk of becoming tail-biting victims. No piglets met the predefined humane endpoint criteria, and therefore no animals required euthanasia.

Two piglets were found dead in the pen: one CS piglet at day 16 post-weaning, suspected to have died from cardiac problems, and one CS piglet at day 18 post-weaning, possibly due to intestinal problems.

### 3.2. Provision of Edible Enrichment

The provision of the enrichment material was higher for PP than CS throughout the trial in terms of absolute weight and also between days 0–14 post-weaning in terms of volume provided (Table 3).

### 3.3. Faecal Consistency Score

A clear drop in faecal consistency was observed in the 1st week post-weaning (see Supplementary Figure S2). No differences in faecal consistency among treatments were observed between days 0 and 14 post-weaning (*p* = 0.82; Table 4). Piglets that received PP as enrichment had a significantly improved faecal consistency in the second half of the trial compared with piglets that received CS as enrichment material (*p* < 0.01). Only one piglet was treated for clinical signs (e.g., dehydration) related to post-weaning diarrhoea in the 1st week post-weaning, and another piglet was found dead in the pen at day 18 post-weaning, possibly due to intestinal problems (see Section 3.1).

### 3.4. Piglet Performance

The performance data of the experimental treatments are presented in Table 5. Piglets that received PP had a significantly increased ADFI (+6.3%; *p* = 0.0498) and ADG (+9.7%; *p* = 0.007) between days 0–9 post-weaning, with a numerically improved FCR (−4.1%; *p* = 0.24) in comparison to piglets given CS. This resulted in a significantly higher BW at day 9 post-weaning for PP versus CS piglets (+160 g; *p* = 0.006), but this effect did not remain at day 14 (*p* = 0.20) and day 28 post-weaning (*p* = 0.82). At the end of the trial, there was less variation in BW when piglets were reared on PP versus CS (−1.2%; *p* = 0.04), which seemed already numerically present at day 14 post-weaning (−1.0%; *p* = 0.16). No effect of edible substrate was observed on overall weaner pig performance between days 0–28 post-weaning (*p* > 0.10).

### 3.5. Explorative and Manipulative Behaviour

**Figure 2:**
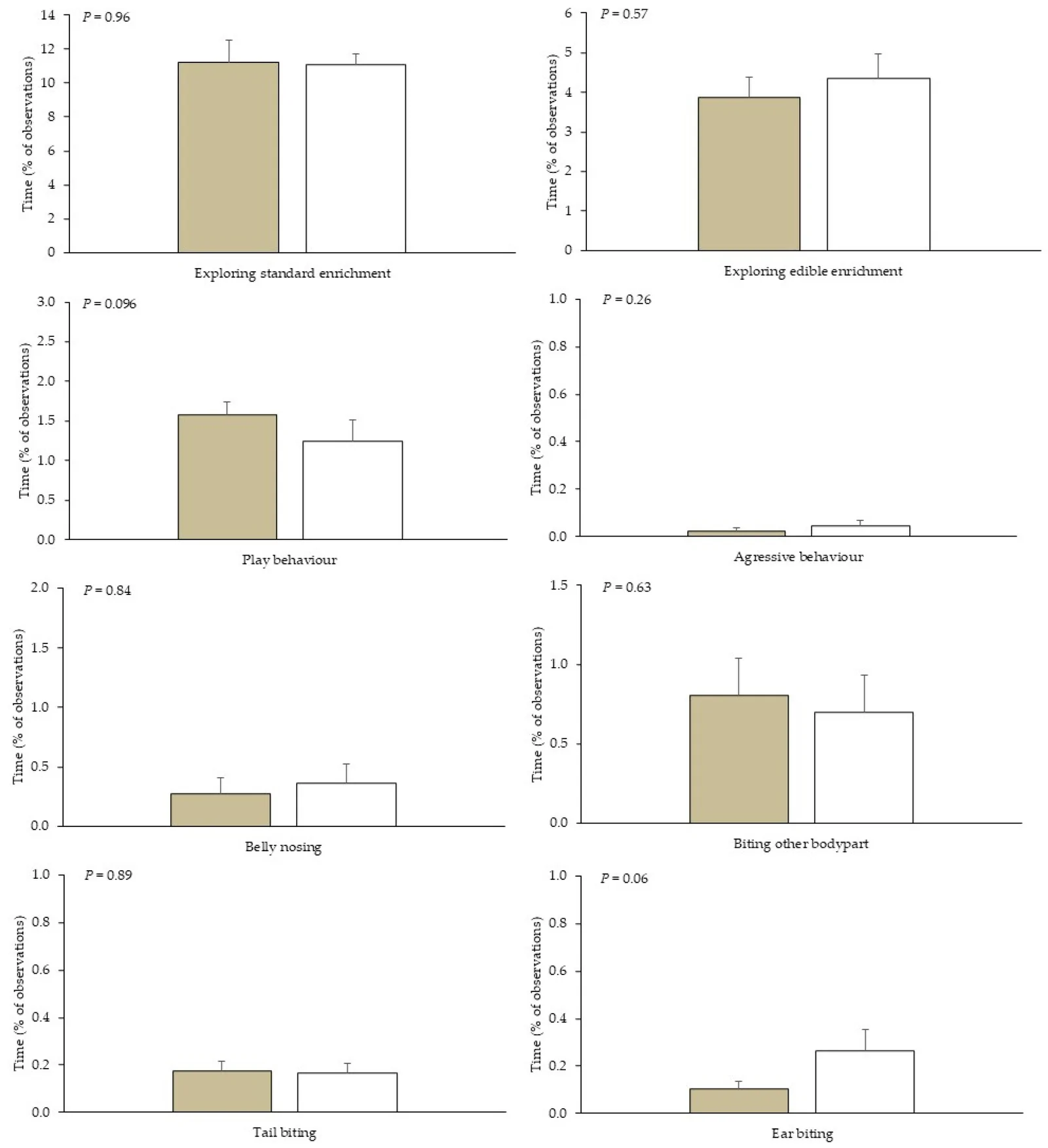
Behavioural activities (% of total observations) at day 8 post-weaning of weaned piglets reared on peat pellets (brown bars) or chopped straw (white bars). Data are presented as absolute means and standard error of the mean (SEM). Statistical analyses were performed on square-root-transformed data.

**Figure 3:**
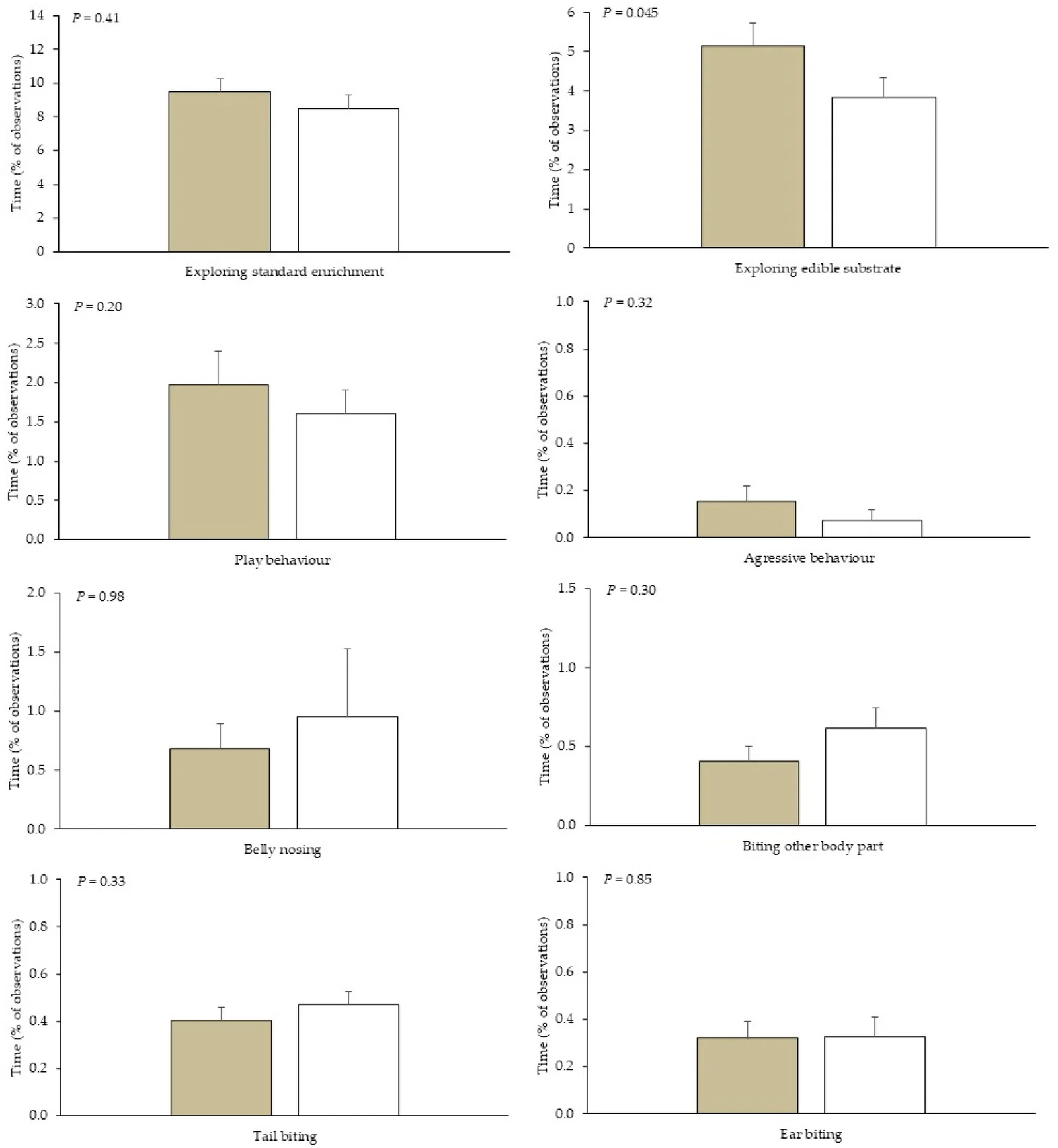
Behavioural activities (% of total observations) at day 18 post-weaning of weaned piglets reared on peat pellets (brown bars) or chopped straw (white bars). Data are presented as absolute means and standard error of the mean (SEM). Statistical analyses were performed on square-root-transformed data.

The behavioural activity of piglets at day 8 and day 18 post-weaning is visualised in Figure 2 and Figure 3, respectively. PP piglets showed numerically more play behaviour (1.58% vs. 1.24%; *p* = 0.096) than CS piglets at day 8 post-weaning. However, this difference was no longer apparent when the complete dataset was analysed (1.45% vs. 1.42%; *p* = 0.81), indicating that the result was sensitive to the outlier-screening procedure. PP piglets tended to perform less ear biting (0.10% vs. 0.26%; *p* = 0.06) than CS piglets at day 8 post-weaning, and PP piglets spent significantly more time on exploring the edible enrichment available (5.15% vs. 3.85%) than CS piglets (*p* = 0.045) at day 18 post-weaning. Explorative behaviour towards the standard pen enrichment, aggressive behaviour, tail biting, biting other body parts, and belly nosing were not affected by treatments.

### 3.6. Tail Damage and Interventions

**Figure 4:**
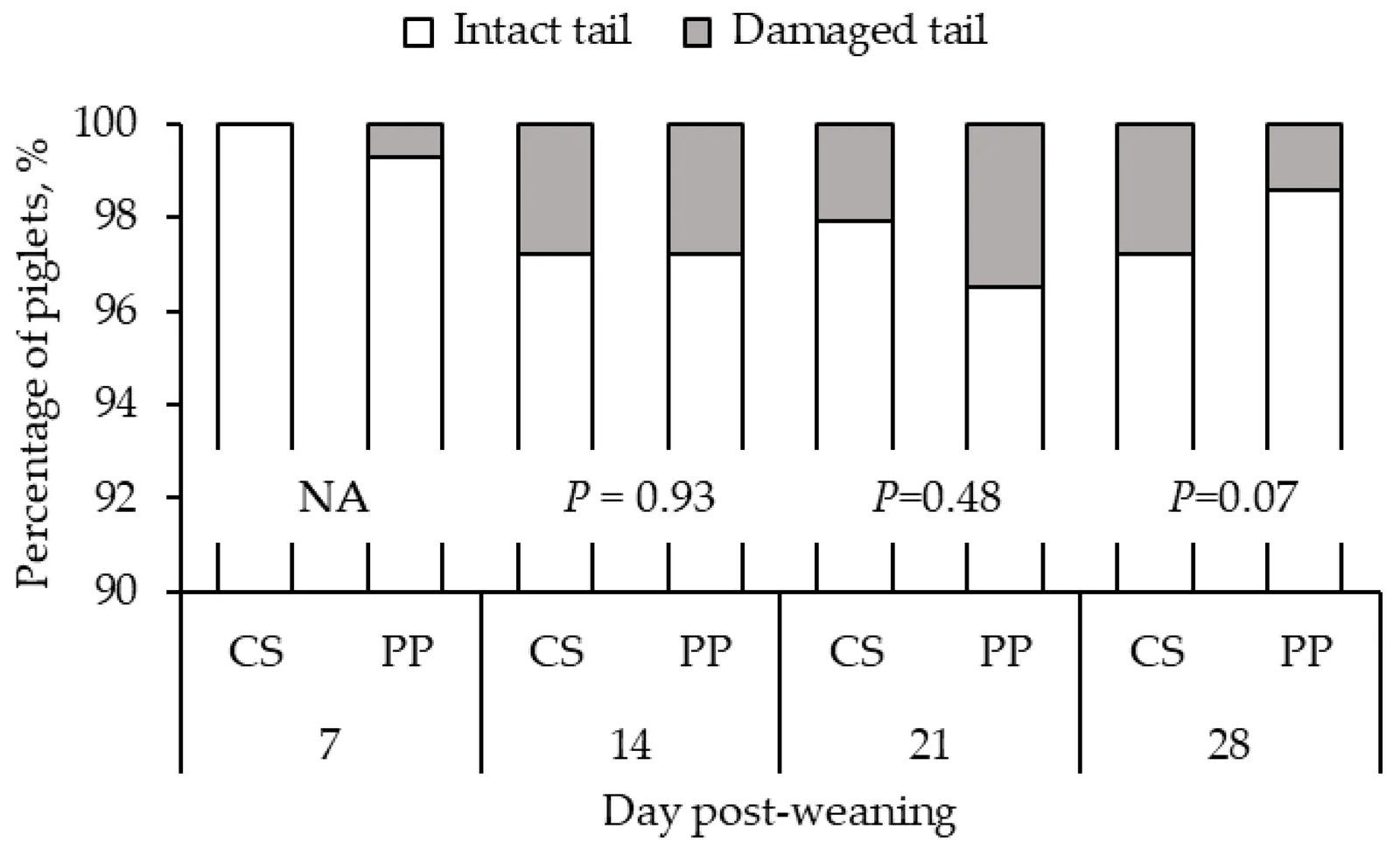
The effect of pen enrichment on the weekly prevalence of tail damage.

Throughout the trial, only ear damage score 0, tail damage score 0, and tail damage score 1 were observed. Ear damage was therefore not statistically analysed. Figure 4 presents the prevalence of tail damage for piglets reared on PP or CS. No statistical analyses on tail damage could be performed at day 7 post-weaning as only one piglet showed tail damage. No effect of edible enrichment type was found on tail damage at day 14 (F_1,11_ = 0.01, *p* = 0.93) or day 21 post-weaning (F_1,11_ = 0.54, *p* = 0.48). Compared with CS, the odds ratio for tail damage in PP piglets was 1.05 (95% CI: 0.36–3.07) at day 14 and 1.74 (95% CI: 0.39–7.75) at day 21 post-weaning. At day 28 post-weaning, PP piglets tended to have lower odds of tail damage than CS piglets (OR = 0.44, 95% CI: 0.20–1.00), corresponding to incidences of 1.4% and 2.9%, respectively (F_1,11_ = 4.06, *p* = 0.07).

A total of 3 out of 24 pens, i.e., 12.5%, required intervention to control tail biting during the trial. The intervention protocol was applied twice during days 0–14 of the trial and twice during days 14–28 of the trial. Interventions were applied to prevent manipulative behaviour towards piglets with mobility problems as a result of meningitis (see Section 3.1 for the removed piglets as result of meningitis), as well as interventions were applied when no clear victim or biter was present, but tail damage was observed on one or more piglets within the pen. The intervention protocol (either removal of the victim or adding a jute bag) was successful as the first day of intervention was sufficient in the pens to prevent fresh blood in the following days. In pen 1.20.5 (i.e., batch.room.pen), the intervention protocol was applied twice, once due to a victim and once due to manipulative biting in the pen. Before analyses of the effect of edible enrichment type on the number of tail biting interventions, the interventions due to a sick victim in the pen were excluded. As only one pen from the PP treatment and one pen from the CS treatment required intervention due to manipulation within the pen to prevent a tail biting outbreak, no statistical analyses could be performed.

## 4. Discussion

We hypothesised that supplementation of PP as enrichment material for weaned piglets with undocked tails would improve growth and faecal consistency, increase exploratory and play behaviours in piglets, and reduce manipulative behaviours directed towards pen mates and the associated body damage.

The positive effects of PP on piglet performance between days 0–9 post-weaning suggest that PP may facilitate adaptation to the weaning transition. The absence of effects later in the post-weaning period indicates that these benefits may be most relevant immediately after weaning. In future studies, it is recommended to measure piglet performance also during the first few days post-weaning (e.g., days 0 to 3) to determine whether PP may be particularly beneficial in helping piglets cope with the weaning transition. Positive zootechnical effects of PP are supported by previous animal performance trials. Supplementation with PP resulted in growth benefits in a 4-week post-weaning trial in piglets with undocked tails [18]. In addition, BW improvements in broiler chickens were previously seen as a result of the same peat product but fed as ingredient mixed into the pelleted diet [17]. As weaning age influences post-weaning adaptation, behaviour, and performance [26], the responses observed in this study may not fully reflect those of piglets weaned at younger or older ages than the piglets included in the current study (30 days of age). For example, the positive effects of peat pellets on feed intake could be more pronounced in earlier-weaned piglets with less experience consuming solid feed before weaning.

The effect of PPs on ADFI between days 0–9 post-weaning may be related to the size of the PP, which concerned 8 mm in diameter and 10–20 mm in length. Chewing on the large diameter PPs may have stimulated motor skills, mastication muscles, and teething of piglets, which are factors involved in the development of feeding behaviour [27]. Studies with large diameter feed pellets have shown that a large diameter improved the feed intake of piglets versus a small diameter [28,29]. The effect was most pronounced during the early feed intake period, when piglets were still adapting to solid feed. This could explain the absence of an effect on ADFI after day 9 post-weaning in the current study. This would be in line with studies reporting increased post-weaning feed intake in newly weaned piglets offered a large diameter pellet (improved ADFI first week post-weaning; 12.7 vs. 3.2 mm in Clark et al. [28]; improved ADFI first 9 days post-weaning; 12 vs. <5 mm in Hewitt et al. [29]). An alternative hypothesis is that offering dietary diversity, by offering two nutritional sources simultaneously instead of one, may enhance feed intake of piglets [30], although this was not investigated in the present study. The feed intake effect of PP provision observed in newly weaned piglets suggest that suckling piglets may also benefit from access to PPs. This hypothesis is supported by previous findings showing that the effects of providing large pellets were more pronounced before than after weaning [31]. In addition to its potential nutritional contribution, PPs may also provide environmental enrichment and stimulate exploratory behaviour before weaning. Therefore, future research should evaluate the effects of pre-weaning exposure to PPs and its potential consequences for adaptation during the weaning transition.

PPs reduced the variation in BW at day 28 post-weaning versus the provision of CS. This is beneficial for pig farmers, as piglets can be reared and sold more homogenously [32]. A second nutritional source for weaned piglets (PPs have a higher nutritional value than CS), next to their main weaner diet, may play a role in this, especially in case of limited feeder access (i.e., 12 piglets/pen with 3 feeding places at the feeder). In case the feeder with commercial diet was occupied, piglets may have redirected themselves to the bowl with PP (with 10 feeding places) to meet their nutritional requirements until the feeder was available again, ensuring a more stable feed intake pattern, which in turn may potentially explain the significantly improved faecal score in the second experimental phase [23]. This hypothesis warrants testing using behavioural observations with a more comprehensive ethogram. Moreover, (inert) fibres and humic acids in PP are expected to modify or stabilize the gut microbiota in the large intestine to affect intestinal fermentation production, and to lower the pH by an increased production of short-chain volatile fatty acids and a reduced production of branched-chain volatile fatty acids [16,17], which in turn protects the intestinal mucosa (reviewed by [33]). The limited fermentation capacity of newly weaned piglets [34] or the higher ADSP, and thus bioactive compounds from PP in the intestine, between days 14–28 versus days 0–14 post-weaning, may explain why an improved faecal score versus CS was only present in the second phase of the experiment. Although different response variables were measured, the improved faecal scores observed in the current study are in line with earlier field observations showing a lower prevalence of post-weaning diarrhoea in pigs provided with PP [18].

The ADSP of PP was 23 g/piglet/d between days 0–14 post-weaning and 37 g/piglet/d between days 14–28 post-weaning. For edible enrichment material, at least 20 g/piglet/d is required to be provided according to Dutch recommendations [35]. In addition, significantly larger amounts of PPs in terms of weight in comparison to CS were provided, while the volume provided was greater during days 0–14 post-weaning. These findings indicate that PPs seem suitable as edible enrichment materials for weaned piglets. Based on a previous trial [18], it was expected that PP would be provided in amounts of approximately 2.5% of the feed intake of weaned piglets. With an ADFI of 322 and 731 g/piglet/d between days 0–14 and days 14–28 post-weaning, respectively, an ADSP of PP of 8 g and 18 g/piglet/d would have been expected. PPs were provided in larger quantities than expected, with 7.1% and 5.1% of the ADFI. It should be noted that part of the recorded provision of the substrate materials may have consisted of losses through the slatted floor. However, the proportion of material lost through the slatted floor could not be quantified. Next to documentation of substrate provision in term of quantity, also behavioural observations suggest that PPs can be used as suitable edible enrichment material when tested against CS. No differences were observed in time spent exploring PP versus CS at day 8 post-weaning, whereas piglets did spend more time exploring PPs than CS at day 18 post-weaning.

As the provisioning level of PP was larger than CS with similar behavioural exploration rates towards the substrate in the first phase of the experiment, it is suggested that the increased level of exploration in the second phase of the experiment may be due to promotion of exploratory and play behaviour towards the substrate rather than an increased level of feeding behaviour towards the substrate (as chewing and nibbling on the substrate was included in the ethogram, which can be followed by ingestion of the substrate). While the greater provision of PP should be interpreted in light of the substantial difference in volume weight between PP and CS, the longer occupancy time observed for PP provides further evidence that piglets were more engaged with this substrate. Previous studies with large diameter feed items confirm that large feed items initiate a greater level of exploratory interaction and object play in piglets versus small pellets [29,30,36]. Although the underlying mechanisms remain unclear, this increased exploration may be related to the easier manipulation of larger feed items, differences in palatability, or their potential to facilitate teething and stimulate play behaviour.

Play is often seen as an indicator of a positive emotional state (e.g., [37]) and a positive welfare indicator (e.g., [38]). PPs tended to stimulate play behaviour of weaned piglets at day 8 post-weaning versus CS. However, the tendency was not observed when all observations were retained in the statistical analysis. Therefore, this non-robust finding should be interpreted with caution. Play observations covered locomotory and social play behaviour, like running across the pen, nudging, and play fighting. Other data collected in this trial suggest that PP may improve this type of play behaviour by stimulating feed intake in the early post-weaning, which may reduce stress of newly weaned piglets (weaned piglets in stress show less play behaviour [39]) and improve the energy balance and growth of the weaned piglet (leaving more energy for play behaviour and better nourished piglets show more locomotion play [40]).

Whether an intervention (e.g., providing substrate, adjusting ventilation or stocking density) is effective in reducing manipulative behaviour and associated damage is dependent on the prevalence of damage [41]. No ear damage occurred in the current trial and the prevalence of tail damage (<5% throughout the trial), and subsequent interventions to prevent further damage, was low for weaned pigs with intact tails [7,42]. Consequently, the statistical power to detect treatment differences for this variable was limited. The low occurrence of damage in the trial may be related to several factors [43], such as farm management, amongst which available (feeder) space per piglet [23], outdoor temperature, and the fact that the edible substrate was provided ad libitum in a feeding bowl, rather than a handful of material twice a day provided on the floor, which is expected to result in more losses of the substrate than in a bowl. The tendency of PP to reduce tail damage incidence at day 28 post-weaning and time spent on ear biting at day 8 post-weaning should be taken with care, as tail damage was observed in only two PP and four CS piglets, while ear biting behaviour accounted for less than 0.5% of the observation time. No differences among treatments were found regarding the time spent on tail biting, which covered only a small proportion of the time of less than 1%. A limitation of the present study is that inter-observer reliability was not assessed. While potential observer bias was reduced by balancing observers across treatments and observation times and rotating observers between rooms every hour, agreement among observers could not be formally quantified. Consequently, caution is warranted when interpreting infrequently observed behaviours, such as ear- and tail-biting behaviour, for which scoring may be more susceptible to observer-related variation. Given the low incidence of tail and ear biting and associated damage observed in the present study, conclusions regarding the effectiveness of PP in preventing damaging behaviours should be drawn with caution. Future studies in higher-risk commercial settings or with a larger sample size are needed to increase the statistical power for welfare-related outcomes such as tail damage. The present study, although results should be taken with care due to a low tail damage incidence and the wide confidence intervals associated with the estimated treatment effect, is consistent with previous farm observations regarding tail biting incidence [18] and suggest PP may facilitate the transition from tail docked to undocked pigs in pig production. Given that tail-directed behaviour can arise in suckling piglets [44], further research is warranted to evaluate the provision of PP in the farrowing room as an early preventive strategy to reduce (later life) damaging behaviour.

The interpretation of the effects of peat pellets is limited in the current study by the inability of the study design to separate nutritional effects from enrichment effects. Peat pellets may influence pigs through multiple pathways, including ingestion-related effects on gut function and behaviour-related effects through increased opportunities for exploration. As these pathways were not experimentally disentangled, the mechanisms underlying the observed responses remain uncertain. To clarify the relative importance of these mechanisms, future research should include additional experimental groups in which peat is provided either exclusively as a dietary component (e.g., incorporated into the feed) or primarily as an enrichment substrate (e.g., loose peat). Such an approach would allow for a more direct assessment of the nutritional versus behavioural contributions of the provision of peat pellets. In addition, the study design does not allow differentiation between effects attributable to the enrichment material itself (peat) and those attributable to its physical form (peat pellets). Previous studies have reported behavioural effects of loose peat [19,20], but a loose peat treatment was not included in the present trial. Consequently, it remains unclear whether the observed responses are specific to peat as a material or are influenced by pelletization and the associated differences in, for example, manipulability and consumption patterns. The provisioning method can also play a role in the effectiveness of enrichment materials [45]. For future studies, it is recommended to explore the most effective method of administering peat pellets to piglets across different pig management systems, for example, mixing the PP through the diet, provision of PP in a (familiar) bowl, or provision on the substrate-covered or barren part of the flooring.

## 5. Conclusions

The current experiment tested the effect of peat pellets as edible enrichment material for weaned piglets up to 4 weeks post-weaning and its effect on piglet performance, faecal score, and piglet explorative and manipulative behaviour. The results of this study are in line with previous findings from studies on the effects of peat pellets in weaned piglets. We conclude that peat pellets are a suitable edible enrichment material for weaned piglets under the conditions of the present study, in which piglets received on average approximately 30 g peat pellets per piglet per day. A greater volume of peat pellets than chopped straw was provided during the first phase of the experiment to ensure continuous availability of the substrate, and piglets exhibited a higher level of exploratory behaviour towards peat pellets than towards chopped straw during the second phase of the experiment. Improvements in piglet performance (ADFI and ADG) were obtained in the early post-weaning period due to provision with peat pellets, while an improved faecal score was observed in the second phase of the experiment, and a reduced variation in BW at the end of the trial within the group provided with peat pellets. The present study was not designed to separate nutritional from enrichment-related mechanisms, nor to distinguish between effects attributable to the peat material itself and those related to its pelletized form and size. A dual role is suggested for peat pellets in pig production, showing positive effects on zootechnical performance (feed intake and growth), while indications were observed for potential welfare-relevant effects (improved faecal consistency). Numerical reductions in ear-biting behaviour and tail damage were also observed. However, given the low incidence of these outcomes and the absence of statistically significant treatment effects, these findings should be considered exploratory. The observed effects should be validated in commercial production systems characterized by a higher incidence of damaging behaviours and weaning ages that differ from those used in the present study.

## Figures and Tables

**Table 1:** The enrichment material provided to weaned piglets in the two treatment groups.

Treatment	Description	Edible Enrichment	Standard Enrichment
PP	Peat pellets	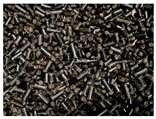	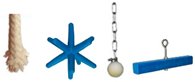
CS	Chopped straw	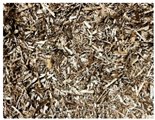	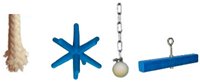

**Table 2:** Definitions of the behavioural categories recorded during observations of piglets after weaning.

**“Explorative Behaviour”**	
Exploring standard enrichment	Sniffing, touching with snout, rooting, flipping over, rolling over floor, pushing, chewing, or nibbling on standard enrichment object or (energetically) shaking head with standard enrichment object in mouth.
Exploring edible enrichment	Sniffing, touching, rooting, flipping over, rolling, pushing, chewing, eating, or nibbling on the peat pellets or chopped straw or (energetically) shaking head with peat pellets or chopped straw in mouth (Note: if these behaviours were directed towards the feeding bowl rather than the edible substrate this did not count and was not recorded).
Play behaviour	Play activities that involve one or more players: running across pen, rolling, pivoting, tossing head, flopping, sliding, scampering, nudging, play fighting.
**“Damaging Behaviour”**	
Tail biting	Chewing, nibbling, or sucking the tail of a pen mate
Ear biting	Chewing, nibbling, or sucking the ear of a pen mate (excluding ear tag)
Belly nosing	Rubbing belly of a pen mate with ≥3 up and down movements of the snout or sucking the navel or skin of the abdominal area of a pen mate
Biting other body part	Chewing, nibbling or sucking part of the body of a pen mate excluding ear, tail, and abdominal area
Aggressive behaviour	Aggressively ramming, pushing, head-knocking, lifting, or biting a pen mate, including mutual fighting
Feed-related fighting	Aggressively ramming, pushing, head-knocking, lifting, or biting a pen mate at the feeder, including mutual fighting for feed at the feeder

The ethogram is based on Middelkoop et al. [24,25]. Biting occurring during agonistic interactions was classified as aggressive behaviour, whereas non-aggressive chewing, nibbling or sucking was classified as biting behaviour.

**Table 3:** The effect of pen enrichment on average daily substrate provision (ADSP) between days 0–28 post-weaning.

	Peat Pellets	Chopped Straw	SEM	*p*-Value
Average daily substrate provision, g/piglet/d (weight provided)				
Day 0–14	22.8 ^b^	2.5 ^a^	0.23	<0.001
Day 14–28	37.4 ^b^	4.7 ^a^	0.31	<0.001
Day 0–28	29.6 ^b^	3.5 ^a^	0.32	<0.001
Number of cups per pen per day (volume provided)				
Day 0–14	3.5 ^b^	3.1 ^a^	0.10	0.04
Day 14–28	5.0	5.0	0.12	0.89
Day 0–28	4.1	4.1	0.12	0.91

Different superscripts (^ab^) within a row indicate a significant difference between treatments (*p* < 0.05). Each treatment consisted of 12 pens with 12 piglets/pen. Values represent pen means. PP had a volume weight of 640 g/L and CS of 80 g/L. The amount of PP and CS provided was recorded daily. As material lost through the slatted floor could not be quantified, actual intake could not be determined.

**Table 4:** The effect of pen enrichment on faecal consistency between days 0–28 post-weaning.

	Peat Pellets	Chopped Straw	SEM	*p*-Value
Day 0–14	5.71	5.75	0.105	0.82
Day 14–28	6.24 ^b^	5.99 ^a^	0.053	0.007
Day 0–28	5.98	5.87	0.067	0.28

Different superscripts (^ab^) within a row indicate a significant difference between treatments (*p* < 0.05). Each treatment consisted of 12 pens with 12 piglets/pen. Faecal consistency was assessed per pen using the scoring system described by Guan et al. [22], with scores ranging from 2 to 9, where the lowest score represents severe watery diarrhoea and the highest score represents hard, dry, and lumpy faeces. Values represent pen means.

**Table 5:** The effect of pen enrichment on piglet performance between days 0–28 post-weaning.

	Peat Pellets	Chopped Straw	SEM	*p*-Value
Body weight (BW), kg				
Day 0	8.27	8.27	0.001	0.30
Day 9	9.79 ^b^	9.63 ^a^	0.034	0.006
Day 14	11.50	11.31	0.098	0.20
Day 28	19.17	19.10	0.223	0.82
Coefficient of variation (CV) in body weight, %				
Day 0	1.4	1.3	0.06	0.20
Day 9	6.8	7.1	0.45	0.67
Day 14	7.4	8.4	0.44	0.16
Day 28	10.7 ^a^	11.9 ^b^	0.36	0.04
Average daily gain (ADG), g/piglet/day				
Day 0–14	231	217	7.0	0.20
Day 0–9	170 ^a^	155 ^b^	3.2	0.007
Day 9–14	333	348	9.2	0.29
Day 14–28	546	556	11.4	0.56
Day 0–28	389	384	7.9	0.68
Average daily feed intake (ADFI), g/piglet/day				
Day 0–14	322	311	5.2	0.19
Day 0–9	235 ^b^	221 ^a^	4.6	0.0498
Day 9–14	476	481	10.6	0.74
Day 14–28	731	743	14.9	0.60
Day 0–28	526	529	9.1	0.84
Feed conversion ratio (FCR), g/g				
Day 0–14	1.40	1.44	0.030	0.36
Day 0–9	1.40	1.46	0.037	0.24
Day 9–14	1.46	1.40	0.025	0.13
Day 14–28	1.34	1.34	0.011	0.89
Day 0–28	1.35	1.38	0.015	0.24

Different superscripts (^ab^) within a row indicate a significant difference between treatments (*p* < 0.05). Each treatment consisted of 12 pens with 12 piglets/pen. Values represent pen means.

## Data Availability

The data presented in this study are available on request from the corresponding author due to the proprietary nature of the data.

## Supplementary Materials

The following supporting information can be downloaded at: https://www.mdpi.com/article/10.3390/ani16203214/s1, Supplementary Dataset; Table S1: Composition and chemical properties of ProHumi^®^ Farm peat pellets (Hankkija Oy, Hyvinkää, Finland); Table S2: Faecal consistency scoring protocol for weaned piglets; Figure S1: Distribution of treatments over the weaner rooms in the experiment; Figure S2: Faecal consistency score of weaned piglets over the post-weaning period, visualised for the two treatment groups. Faecal consistency was registered on an 8-point scale from sever water thin diarrhoea (score 2) to hard, dry, and lumpy faeces (score 9). Faecal scoring took place three times a week, resulting in 12 measures per pen.

## Abbreviations

The following abbreviations are used in this manuscript:

PP	Peat pellets
CS	Chopped straw
ADSP	Average daily substrate provision
