# Supplementary material for: Peat Pellets as Enrichment Material for Weaned Piglets
Source: Animals (Basel). 2026 Oct 13;16(20):3214. doi: 10.3390/ani16203214 (PMC13700616; doi:10.3390/ani16203214)
Supplement: Supplementary file 1 [file animals-16-03214-s001.zip › Supplementary material to Article Peat Pellets.pdf]

Supplementary material to Article

Peat pellets as enrichment material for weaned piglets

Anouschka Middelkoop, Hannele Kettunen, Anne Maria Stevina Huting, Juhani Vuorenmaa, and Francesc Molist

Animals, 2026.

Table S1. Composition and chemical properties of ProHumi® Farm peat pellets (Hankkija Oy, Hyvinkää, Finland)

**Ingredients**	
100% selected, natural, heat-treated peat from Finland	
**Chemical properties**	
Moisture	12.0%
Ash	< 2.5%
Crude fibre	Min. 16.0%
Dietary fibre	71.3%
NDF	63.6%
ADF	54.6%
ADL	44.7%
Humic acids	25-30.0%

Table S2. Faecal consistency scoring protocol for weaned piglets

**Score**	**Description**
2	Water thin faeces. Flows through slatted floor.
3	Liquid faeces. Flows through slatted floor.
4	Loose faeces, like custard.
5	Smooth, shapeless pile of faeces.
6	Mushy faeces without structure, like peanut butter.
	Sticks to glove when picked up.
7	Firm and shaped faeces without structure (smooth).
8	Firm and shaped faeces with structure (cracks on the surface).
	Able to be picked up as a whole.
9	Hard, dry and lumpy faeces.
	After being picked up, the glove is not or hardly dirty. Falls apart after shaking.

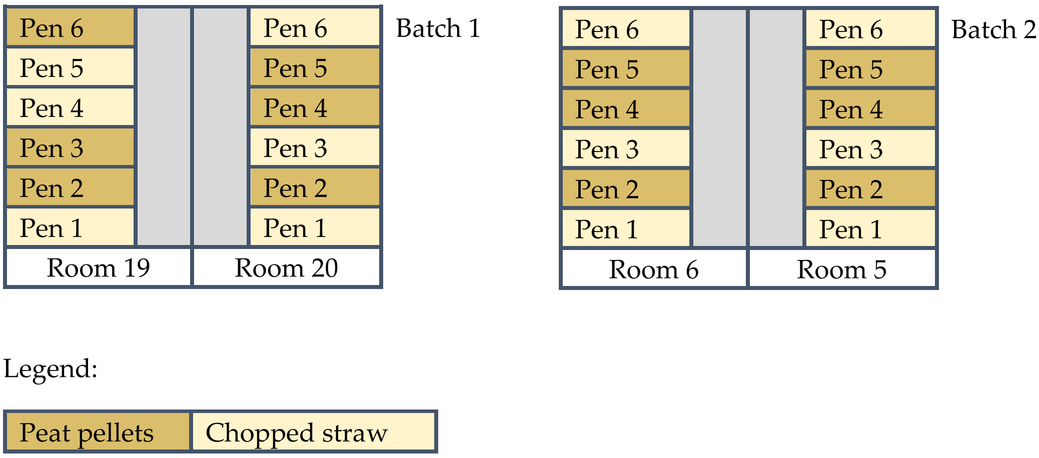

Figure S1. Distribution of treatments over the weaner rooms in the experiment.

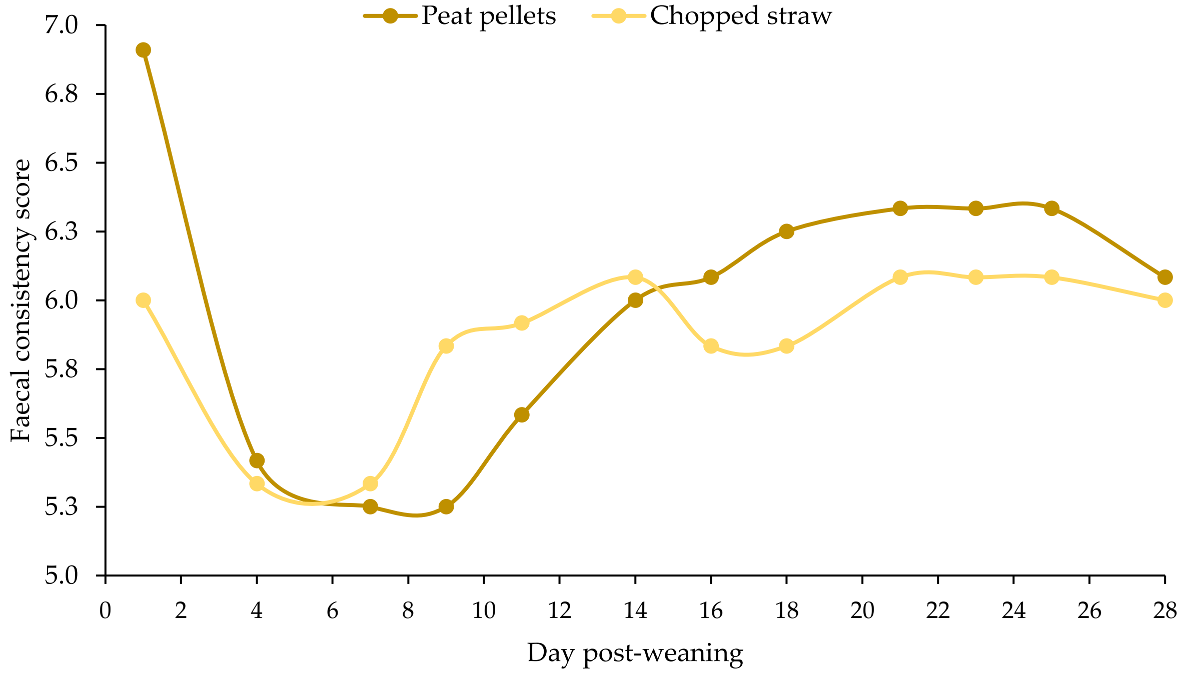

Figure S2. Faecal consistency score of weaned piglets over the post-weaning period, visualised for the two treatment groups. Faecal consistency was registered on an 8-point scale from sever water thin diarrhoea (score 2) to hard, dry, and lumpy faeces (score 9). Faecal scoring took place three times a week, resulting in 12 measures per pen.
